# Novel Respiratory Virus Infections in Children, Brazil

**DOI:** 10.3201/eid1505.081603

**Published:** 2009-05

**Authors:** Maria Carolina M. Albuquerque, Gisele P.A. Pena, Rafael B. Varella, George Gallucci, Dean Erdman, Norma Santos

**Affiliations:** Universidade Federal do Rio de Janeiro, Rio de Janeiro, Brazil (M.C.M. Albuquerque, G.P.A. Pena, N. Santos); Centro Universitário Serra dos Órgãos, Teresópolis, Rio de Janeiro (R. B. Varella); Centers for Disease Control and Prevention, Atlanta, Georgia, USA (G. Gallucci, D. Erdman)

**Keywords:** Respiratory infections, viruses, human coronavirus, human bocavirus, human metapneumovirus, human polyomavirus, Brazil, dispatch

## Abstract

Recently discovered respiratory viruses were detected in 19 (9.2%) of 205 nasal swab specimens from children in Brazil with respiratory illnesses. Five each were positive for human metapneumovirus (HMPV) alone and human bocavirus (HBoV) alone, 3 for human coronaviruses (HCoV-HKU1 or -NL63) alone, and 6 for more than 1 recently discovered virus.

Viral infections are among the leading causes of respiratory disease in children. Most of these infections are caused by respiratory syncytial virus (RSV), influenza virus A or B (FluV), parainfluenza virus (PIV), rhinovirus (RV), or adenovirus (AdV). Several recently discovered viruses, such as human metapneumovirus (HMPV), human bocavirus (HBoV), and the human coronaviruses (HCoVs) NL63 and HKU1, have been identified as potential respiratory pathogens (*1*). In addition, 2 new human polyomaviruses (HPyVs), KIPyV and WUPyV, have been detected in patients with respiratory infections (*1*). In Brazil, epidemiologic studies have demonstrated the extent to which viruses cause respiratory illness in children (*2*–*4*). However, because such studies have focused on the most common viral pathogens, the extent to which the novel respiratory viruses are etiologic agents of respiratory disease in Brazilian children remains unknown. In this study, we sought to investigate the occurrence of respiratory infections associated with HMPV, HBoV, HCoV-HKU1, HCoV-NL63, KIPyV, and WUPyV among children in Brazil.

## The Study

The study protocol was reviewed and approved by the research ethics committees of the Institute of Puericulture and Pediatrics Martagão Gesteira of the Federal University of Rio de Janeiro and the Educational Foundation of Serra dos Órgãos of Teresópolis. The parents of all children involved in the study gave informed consent for their children’s participation in accordance with Resolution 196/96 of the Brazilian Ministry of Health.

Nasal swabs from 205 children (median age 3.3 years; range 1 month to 15 years) with acute upper or lower respiratory illnesses were collected from March 2006 through October 2007 and tested for viral pathogens. Acute respiratory illness was defined by the presence of rhinorrhea, cough, respiratory distress, or sore throat, associated or not with fever, for a maximum duration of 7 days. The specimens were collected from hospitalized patients, emergency departments, and walk-in clinics at 2 university hospitals in the cities of Rio de Janeiro and Teresópolis. Relevant clinical information, including patients’ hospitalization status, age, sex, and clinical symptoms, was collected during the first medical visit by means of a standard questionnaire.

The nasal swabs were immersed in 1 mL of virus transport media and kept at –70^o^C until processing. Nucleic acid was extracted from 200 µL of the sample by using the Wizard Genomic DNA Purification kit (Promega, Madison, WI, USA) or RNAgents kit (Promega) according to the manufacturer’s instructions. Specimens were tested for presence of FluV A and B (*5*), PIV 1- 4 (*6*), AdV (*7*), RSV (*8*), RV (*8*), HMPV (*9*), HBoV (*10*), WUPyV (*11*), and KIPyV (*12*) by conventional PCR assays as previously described. A real-time PCR protocol was used for detection of HCoVs (229E, OC43, NL63, and HKU1) (*13*).

Of the 205 samples tested, 63 (30.7%) were positive for at least 1 of the viral pathogens specified above. Nineteen (9.2%) were positive for at least 1 of the newly described viruses: 5 for HMPV only, 5 for HBoV only, 3 for HCoV-HKU1 or HCoV-NL63 only, and 6 for co-infections with these viruses, including 2 samples positive for KIPyV or WUPyV. Of the samples positive for common respiratory viruses, 33 were positive for rhinovirus only, 5 for FluV A only, 3 for RSV only, and 1 each for HCoV-OC43 and AdV only. Two samples were positive for >1 common respiratory viruses, and PIV was not detected (Appendix Table). The age of the patients infected with the newly described viruses ranged from 4 months to 11 years (median 2.7 years). The most frequent clinical symptoms were fever, rhinorrhea, cough, sore throat, wheezing, bronchiolitis, and pneumonia (Table). Although the specimens were collected from symptomatic children and a wide range of viruses were screened for by highly sensitive methods, less than one third of the samples were positive. Perhaps if we had collected nasopharyngeal swabs or aspirates, the percentage of samples that tested positive would have been higher.

**Table:** Clinical symptoms observed among patients with a single infection, by virus, Brazil, 2006–2007*

Clinical symptom	% Patients (no. positive/no. tested)							
	HCoV-HKU1 or NL63	HMPV	HBoV	RV	FluV A	RSV	HCoV-OC43	AdV
Fever	100 (3/3)	80 (4/5)	80 (4/5)	52 (17/33)	40 (2/5)	100 (3/3)	0 (0/1)	100 (1/1)
Rhinorrhea	33 (1/3)	60 (3/5)	60 (3/5)	67 (22/33)	80 (4/5)	100 (3/3)	0 (0/1)	0 (0/1)
Cough	33 (1/3)	100 (5/5)	80 (4/5)	67 (22/33)	40 (2/5)	33 (1/3)	0 (0/1)	100 (1/1)
Sore throat	0 (0/3)	20 (1/5)	20 (1/5)	6 (2/33)	60 (3/5)	0 (0/3)	0 (0/1)	0 (0/1)
Wheezing	33 (1/3)	40 (2/5)	80 (4/5)	27 (9/33)	20 (1/5)	0 (0/3)	0 (0/1)	100 1/1)
Bronchiolitis	33 (1/3)	20 (1/5)	0 (0/5)	12 (4/33)	0 (0/5)	0 (0/3)	100 (1/1)	0 (0/1)
Pneumonia	100 (3/3)	0 (0/5)	40 (2/5)	27 (9/33)	20 (1/5)	0 (0/3)	0 (0/1)	0 (0/1)

*HCoV, human coronavirus; HMPV, human metapneumovirus; HBoV, human bocavirus; RV, rhinovirus; FluV A, influenza virus A; RSV, respiratory syncytial virus; AdV, adenovirus.

The diagnosis for most patients infected with HCoV-NL63 or HCoV-HKU1 was pneumonia. Of the 3 patients with single infections, 2 were hospitalized and 1 was treated at the emergency department; the 2 patients with mixed infection of HCoV-NL63 and HMPV or RV were treated at walk-in clinics.

Patients with HMPV infections had a myriad of symptoms, including fever, cough, rhinorrhea, wheezing, and sore throat. Of the 5 patients with single infections, 4 were treated at walk-in clinics and 1 was treated at an emergency department. A patient co-infected with HMPV and HCoV-NL63 was treated at a walk-in clinic, and a patient co-infected with HMPV and KIPyV and 1 co-infected with HMPV, HCoV-OC43, AdV, and RV were treated at an emergency department. The patient co-infected with KIPyV was a 4-year-old boy with cough, fever, rhinorrhea, and wheezing.

HBoV was detected in samples from 5 patients as a single infection and in samples from 2 patients as a co-infection with RV or WUPyV. Three patients had pneumonia (2 single infections and 1 co-infection with RV). Two of the 3 were treated at walk-in clinics; 1 of the patients with only HBoV infection was hospitalized. The patient co-infected with WUPyV was a 10-month-old boy who had been treated at an emergency department after exhibiting cough, rhinorrhea, and laryngomalacia.

## Conclusions

Previous studies have documented the importance of respiratory virus infections among pediatric patients in Brazil (*2*–*4*). However, the effect of the so-called emerging respiratory viruses on the children of Brazil is yet to be clarified. Few studies have demonstrated the circulation of HMPV among Brazilian children (*4*,*14*), and to our knowledge, none have described the circulation of HBoV, HCoV, or HPyVs as respiratory pathogens in Brazil, although 1 study did report the presence of HBoV in the stools of Brazilian children with gastroenteritis (*15*). Our finding that HMPV, HBoV, HCoV-HKU1 or HCoVB-NL63, or the newly described KIPyV or WUPyV was present in 9.2% of the tested samples suggests that these viruses could be important respiratory pathogens in the country. However, further investigative studies that include appropriately matched control groups will be necessary to demonstrate that these novel viruses act as etiologic agents of respiratory disease in Brazil.

## Supplementary Material

Appendix Table Clinical characteristics of patients infected with various respiratory viruses, Brazil, 2006-2007*
