# Supplementary material for: Novel Respiratory Virus Infections in Children, Brazil
Source: Emerg Infect Dis. 2009 May;15(5):806–8. doi: 10.3201/eid1505.081603 (PMC2687000; doi:10.3201/eid1505.081603)
Supplement: Appendix Table — Clinical characteristics of patients infected with various respiratory viruses, Brazil, 2006-2007* [file 08-1603_appT-s1.pdf]

Appendix Table. Clinical characteristics of patients infected with various respiratory viruses, Brazil, 2006–2007*

Virus (no. patients)	Age/sex	Status	Clinical symptoms
Single infections			
HCoV-HKU1 (1)	4 mo/F	H	Pneumonia, bronchiolitis, fever, wheezing, rhinorrhea
HCoV-NL63 (2)	2 y/M	E	Pneumonia, fever
	2 y 5 mo/M	H	Pneumonia, fever, cough
HMPV (5)	6 mo/M	E	Bronchiolitis, fever, cough, vomiting, exanthem
	4 y/M	C	Fever, cough, wheezing, rhinorrhea
	5 y/M	C	Sore throat, cough, rhinorrhea, vomiting
	11 mo/F	C	Fever, cough
	7 mo/F	C	Fever, rhinorrhea, cough, wheezing, vomiting, diarrhea
HBoV (5)	2 y 5 mo/M	C	Pneumonia, fever, cough, wheezing
	1 y/M	C	Fever, cough, rhinorrhea, wheezing, vomiting
	?/M	C	Sore throat, cough, rhinorrhea
	?/M	C	Fever, rhinorrhea
	1 y 8 mo/F	H	Pneumonia, fever, cough, wheezing
RV (33)	1 y/F	C	Cough, rhinorrhea
	10 y/F	C	Fever, vomiting, headache
	3 y/F	E	Fever, cough, rhinorrhea, wheezing
	5 y/F	H	Fever, sore throat, cough, nasal congestion, asthma
	7 y/F	E	Fever, sore throat
	10 y/F	H	Rhinorrhea, asthma
	2 mo/F	C	Cough, rhinorrhea, nasal congestion, wheezing
	5 y/F	C	Cough, nasal congestion, wheezing, asthma
	2 mo/F	E	Fever, nasal congestion
	10 mo/F	E	Fever, nasal congestion, cough, rhinorrhea
	1 y 4 mo/F	C	Pneumonia, bronchiolitis, fever, nasal congestion, cough, wheezing
	1 y/F	E	Pneumonia
	4 y/F	C	Fever, cough, nasal congestion, vomiting
	1 y/F	C	Cough, rhinorrhea, wheezing
	7 mo/F	C	Bronchiolitis, cough, rhinorrhea, wheezing
	8 mo/F	C	Fever, cough, rhinorrhea
	2 y/M	C	Rhinorrhea, vomiting, diarrhea
	6 mo/M	E	Broncospasm
	7 mo/M	H	Fever, cough, nasal congestion, rhinorrhea
	3 mo/M	H	Pneumonia, fever
	9 y/M	E	Cough, rhinorrhea, asthma
	12 y/M	H	Pneumonia, cough, wheezing, rhinorrhea
	1 y/M	C	Pneumonia, cough, rhinorrhea
	7 y/M	C	Pneumonia, cough
	6 y/M	H	Pneumonia, fever, rhinorrhea
	9 mo/M	E	Cough, rhinorrhea
	1 mo/M	H	Pneumonia, broncospasm, fever, rhinorrhea
	9 mo/M	H	Broncospasm, fever, rhinorrhea
	4 mo/M	H	Broncospasm, cough, rhinorrhea, nasal congestion
	10 mo/M	E	Pneumonia, fever, rhinorrhea
	3 y/M	C	Bronchitis, wheezing, cough
	7 mo/M	C	Bronchitis, wheezing, cough, rhinorrhea
	6 y/M	C	Fever, cough, rhinorrhea
	?/M	C	Fever, cough, rhinorrhea
FluV A (5)	15 y/F	C	Sore throat, rhinorrhea, nasal congestion
	1 y/F	C	Fever, cough, rhinorrhea, nasal congestion, wheezing
	9 y/F	C	Sore throat, rhinorrhea
	10 y/F	H	Pneumonia, fever, cough
	2 y/M	H	Sore throat, rhinorrhea, nasal congestion
RSV (3)	9 mo/M	E	Fever, rhinorrhea
	4 mo/M	E	Fever, rhinorrhea
	5 mo/M	E	Fever, cough, rhinorrhea, nasal congestion
HCoV-OC43 (1)	3 mo/F	H	Bronchiolitis
AdV (1)	5 mo/F	C	Fever, cough, nasal congestion, wheezing
Co-infections			
HCoV-NL63 + HMPV	6 y/F	C	Sore throat, cough
HCoV-NL63 + RV	8 y/F	C	Pneumonia, fever, cough
HMPV + RV + HCoV-OC43 + AdV	11 y/F	E	Fever, rhinorrhea

HMPV + KIPyV	4 y/M	E	Fever, cough, rhinorrhea, wheezing
HBoV + RV	1 y/F	C	Pneumonia, fever, cough, wheezing, rhinorrhea
HBoV + WUPyV	10 mo	E	Cough, rhinorrhea, laryngomalacia
FluV A + RV	1 y 4 mo	C	Fever, rhinorrhea, diarrhea, ear pain
RSV + HCoV-OC43	5 mo/M	E	Bronchiolitis, rhinorrhea

*HCoV, human coronavirus; H, hospitalized; E, emergency department; HMPV, human metapneumovirus; C, walk-in clinics; HBoV, human bocavirus; ?, unknown; RV, rhinovirus; FluV A, influenza virus A; RSV, respiratory syncytial virus; AdV, adenovirus; KIPyV and WUPyV, human polyomaviruses.
